# Neurobiological Sex Differences in Developmental Dyslexia

**DOI:** 10.3389/fpsyg.2018.02669

**Published:** 2019-01-11

**Authors:** Anthony J. Krafnick, Tanya M. Evans

**Affiliations:** ^1^Psychology Department, Dominican University, River Forest, IL, United States; ^2^Center for Advanced Study of Teaching and Learning, Curry School of Education and Human Development, University of Virginia, Charlottesville, VA, United States

**Keywords:** dyslexia, sex differences, reading, neuroanatomy, genetics

## Abstract

Understanding sex differences at the neurobiological level has become increasingly crucial in both basic and applied research. In the study of developmental dyslexia, early neuroimaging investigations were dominated by male-only or male-dominated samples, due at least in part to males being diagnosed more frequently. While recent studies more consistently balance the inclusion of both sexes, there has been little movement toward directly characterizing potential sex differences of the disorder. However, a string of recent work suggests that the brain basis of dyslexia may indeed be different in males and females. This potential sex difference has implications for existing models of dyslexia, and would inform approaches to the remediation of reading difficulties. This article reviews recent evidence for sex differences in dyslexia, discusses the impact these studies have on the understanding of the brain basis of dyslexia, and provides a framework for how these differential neuroanatomical profiles may develop.

## Introduction

Developmental dyslexia is a neurodevelopmental learning disability, estimated to affect between 5 and 13% of the U.S. population (Katusic et al., 2001). The hallmark behavioral profile of dyslexia is difficulty with recognition and decoding of words that cannot be accounted for by classroom instruction, motivation, or overall cognitive abilities (Lyon et al., 2003; Peterson and Pennington, 2012). Neuroimaging studies suggest aberrations to temporo-parietal and inferior frontal language regions and occipito-temporal visual processing cortex (e.g., the visual word-form area, VWFA, in the left fusiform; Dehaene et al., 2002) in comparison to age-matched control subjects (Maisog et al., 2008; Richlan et al., 2011, 2013; Linkersdörfer et al., 2012; Eckert et al., 2016; Martin et al., 2016). However, individual studies remain variable in terms of the brain profile of dyslexia. Some of this may be due to the age of participants (Richlan et al., 2011; Martin et al., 2015), the potential for different subtypes of reading difficulty (Tamboer et al., 2015), or more specifically degree of reading experience (Krafnick et al., 2014), but another crucial and often overlooked factor for dyslexia as a field is the potential impact of sex differences.

**FIGURE 1:**
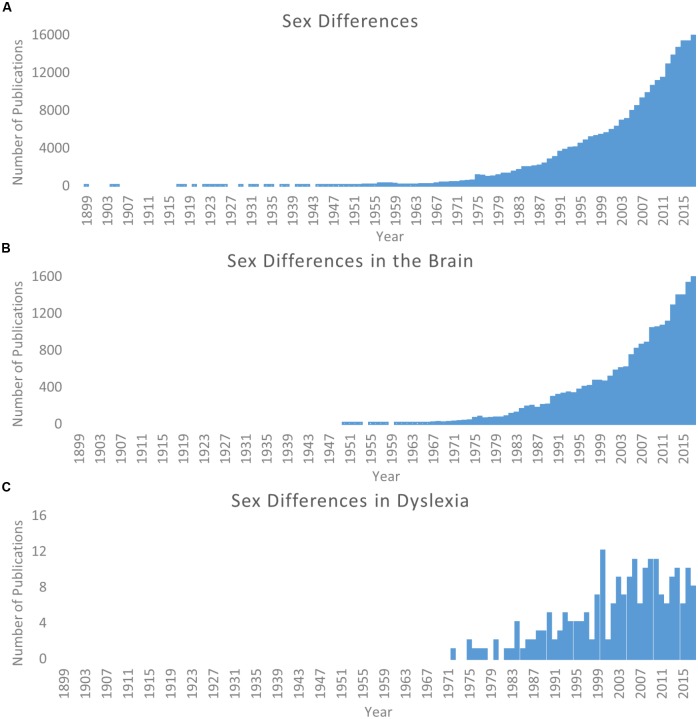
Literature of sex differences over time. Searches via PubMed. **(A)** Results for search parameters: (Sex differences; OR Gender differences). **(B)** Results for search parameters: (Sex differences; OR Gender differences) AND brain. **(C)** Results for search parameters: (Sex differences; OR Gender differences) AND dyslexia. Note scale difference for *y* axes across panels.

The study of sex differences has become an increasingly relevant topic of scientific pursuit. Searching for “sex differences” on PubMed^1^ returns over 250,000 publications from 1899 through 2017, and over half of these occur within the last 10 years (2008–2017; Figure 1A). A similar pattern is observed for the study of sex differences in the brain (Figure 1B). As such, the particular importance of this work for better characterization of the brain and individual differences in behavior has been reviewed and discussed in detail (Cahill, 2006, 2012; Cosgrove et al., 2007; McCarthy et al., 2012; Hausmann, 2017). In terms of human brain structure alone, sex differences are observed within the typical population in children and adolescents (De Bellis et al., 2001; Lenroot et al., 2007; Peper et al., 2009; Simmonds et al., 2014; Tyan et al., 2017), adults (Good et al., 2001; Luders et al., 2009), the elderly (Coffey et al., 1998), and across the lifespan (Sowell et al., 2007; Giedd et al., 2012). Several of these studies capture changes over time in longitudinal samples (Coffey et al., 1998; De Bellis et al., 2001; Peper et al., 2009; Simmonds et al., 2014). These are only but a few examples of the now vast literature; for a recent meta-analysis see Ruigrok et al. (2014).

Beyond the importance of understanding these differences in the typical population is the necessity for this research in order to fully understand the etiology of psychiatric, neurological, and developmental disorders related to brain structure and function (Cahill, 2006; Cosgrove et al., 2007; Hausmann, 2017; Choleris et al., 2018). Acknowledging the existence and importance of potential sex differences can drastically change the understanding of behavior, brain structure, and brain function in models of both typical development and disease (see Cahill, 2012 for examples). This is particularly crucial considering many levels (e.g., rodent, non-human primate, human) of neuroscience research are often dominated by male samples; it is a fallacy to assume these results can simply be extrapolated to the greater population of both sexes (Cahill, 2012; Choleris et al., 2018). Additionally, while studying sex differences in the typical population can provide insight that is relevant to neurological and psychiatric phenomena, it is equally crucial to study sex differences in the context of disorders to obtain a full picture. Sex differences within the context of a disorder may reflect an exaggeration of typical sex differences (e.g., brain differences described above) or help elucidate unique characteristics of sex to the etiology of manifestation of symptoms. Only extending research in both of these domains will provide the critical information needed to determine how sex differences impact specific disorders.

While it is true that evidence for the influence of sex is growing (highlighted by the dramatic increase in studies on the topic), the importance of this work is still not universally embraced by the neuroscience community. In a call to action for brain scientists, Cahill (2006) described several misconceptions that are commonly used to shrug off influences of sex on the brain. These misconceptions include: (1) sex effects are small and unreliable, and (2) if there are no sex differences in behavior than the brain must also show no differences. Over a decade later, there are still periodic calls for the greater neuroscience community to take sex differences into account within their respective domains of study (Cahill, 2012; McCarthy et al., 2012; Choleris et al., 2018), including directly from funding agencies (Clayton and Collins, 2014), because much of this skepticism continues to persist. In the domain of language, while several studies have demonstrated sex differences in brain function for language-related paradigms in both children and adults (Shaywitz et al., 1995; Jaeger et al., 1998; Rossell et al., 2002; Bell et al., 2006; Burman et al., 2008, 2013), there are not necessarily consistent findings for sex differences in language specific regions of cortex (Wallentin, 2009). As the study of sex differences continues to grow, individual fields will be better able to compare across studies and amongst varying paradigms to disentangle where these differences do exist and how they affect our understanding of both the healthy and disordered brain.

The dyslexia neuroimaging field represents an important area of study that needs to take into account the potential for male and female differences. One important indicator is that dyslexia (similar to many other neurodevelopmental disorders, e.g., autism; Halladay et al., 2015) has higher odds ratios for boys compared to girls (Flannery et al., 2000; Katusic et al., 2001; Rutter et al., 2004; Liederman et al., 2005; Quinn and Wagner, 2015). Likely, at least in part due to this discrepancy, the neuroimaging literature has been biased toward higher numbers of male subjects. For example, of the studies included in a meta-analysis of functional differences between individuals with dyslexia and typical controls (Richlan et al., 2011), 65% of the subjects in pediatric studies were male and 95% of the subjects in adult samples were male. A similar trend is observed in structural brain imaging studies: 81% of subjects with dyslexia were male in a meta-analysis of volumetric studies of dyslexia (Richlan et al., 2013).

Considering the odds ratio discrepancy, it is reasonable that there have historically been more male subjects included in these studies. However, this is problematic because (as discussed above) it cannot be assumed females will show the same profile as their male counterparts. Even now, when more studies show similar numbers of males and females in their cohorts, the framework for interpretation still relies on theories generated by male-dominated samples. Since the original call for studying sex differences in neuroimaging studies of dyslexia (Lambe, 1999) there has been an increase in attention (see Figure 1C), but only more recently has there been a noticeable change in specifically looking for potential sex differences. Structural differences between females and males (as compared to typical controls) have been observed (Sandu et al., 2008; Altarelli et al., 2013, 2014; Clark et al., 2014; Evans et al., 2014; Su et al., 2018), and in a separate line of investigation, it has been demonstrated that genetic risk for dyslexia may be mediated in part by estrogen and estrogen receptors (Massinen et al., 2009; Tammimies et al., 2012). As such, Ramus et al. (2018) recently highlight sex as an often overlooked but potential explanatory factor for the heterogeneity of findings in dyslexia imaging studies. Continued work in these domains has great potential impact on understanding the etiology of dyslexia and the manifestation of brain-based differences between individuals with dyslexia and typical readers, as well as in shaping how educators and scientists design interventions to ameliorate reading difficulties.

The purpose of this review is to: (1) highlight recent work demonstrating differences between males and females with dyslexia (2) encourage researchers in the field to not just balance sex inclusion but to make understanding sex differences a specific and purposeful aspect of studies whenever possible, and (3) present a potential framework for the manifestation and relevance of these differences. We first discuss recent evidence for sex differences in dyslexia with a brief section on behavioral evidence, followed by a review of the recent neuroimaging research that speaks to brain differences in males and females with dyslexia. Next, we explore the genetic risk factors for dyslexia that may affect differentiation of the brain basis of dyslexia between the sexes. Finally, we conclude by describing how this recent work impacts our understanding of the dyslexic brain and make recommendations for the field to consider moving forward.

## Behavioral Evidence

Given the differential diagnosis of developmental dyslexia between males and females, one might also expect sex differences in reading behavior to manifest. These differences could present in the performance of dyslexic individuals on measures of either domain-specific abilities of spoken or written language, or more domain-general characteristics (e.g., IQ, working memory, processing speed, inhibitory control) that could provide an alternative explanatory account. Keeping in mind that a divergence in behavior would not necessarily accompany a divergence in etiology for dyslexic males and females (for review see Vogel, 1990), there has been some exploration into sex-specific behavioral differences, especially since there is evidence of such expression in typical readers. Overall, the literature on behavioral sex differences in reading behavior is quite limited, which is not surprising given that diagnosis and evaluation is identical for all children. We provide a brief review of behavioral findings in the general and learning-disabled populations here, and discuss implications for similar behavioral profiles manifesting from differential genetic and neurobiological cascades.

Among typical readers, there is the tendency for girls to acquire reading skills at a more rapid pace than boys (*n* = 87; Wolf and Gow, 1986). This has been attributed to a female advantage in both the rate of linguistic processing as well as in basic word recognition. Across the range of reading abilities (including those at risk for reading difficulty), there is evidence for not only lower mean scores in males, but also a higher variance in reading ability in males compared to females; in the low end of the distribution males are disproportionately represented, but no sex differences are found in the high end (*n* = 2,399; Arnett et al., 2017). Slower processing speed and lower inhibitory control in males provides partial explanation, although males do demonstrate higher verbal reasoning which could enable some degree of compensation. On the whole, differential strengths and weaknesses in both domain-specific reading and domain-general attributes are seen in males and females.

In the realm of disability, school-aged females identified with learning disabilities (LDs), including but not specific to reading disability, demonstrate lower intelligence scores than their male counterparts (Holowinsky and Pascale, 1972; Bradbury et al., 1975; Lawson et al., 1987). Interestingly, this pattern also manifests in young adults (Vogel and Walsh, 1987; *n* = 49). Analogous to what has been reported in typical readers, relative to males with LD, females with LD display strengths in verbal conceptualizations. This is consistent with the observed relative strength in language skills in female children with dyslexia, with dyslexic boys demonstrating lower scores in working memory and orthographic coding compared to the girls (Berninger et al., 2008; *n* = 122). A small (*n* = 7) eye tracking study of visual prediction in dyslexia produced some interesting results in the realm of visuospatial processing: males with dyslexia perseverated on the current target, where females with dyslexia more quickly predicted subsequent targets (Suroya and Al-Samarraie, 2016).

These results as a whole indicate that females with dyslexia may have a combination of strengths in both domain-general (e.g., IQ, working memory, visuospatial) and domain-specific (e.g., verbal conceptualization, orthographic coding) reading skills relative to their male counterparts. On the other hand, males tend to have higher domain-specific skills in verbal reasoning abilities. This line of research supports a differential dyslexic phenotype in males and females, which is further corroborated by results at the brain and genetic levels, detailed below.

## Brain Evidence

### General Neuroanatomy of Dyslexia

The neuroanatomical profile of dyslexia has been under investigation for several decades. From early *post mortem* studies (Galaburda and Kemper, 1979; Galaburda et al., 1985; Humphreys et al., 1990) to more recent neuroimaging meta-analyses (Maisog et al., 2008; Richlan et al., 2011, 2013; Linkersdörfer et al., 2012; Eckert et al., 2016; Martin et al., 2016), the literature suggests a primarily left hemisphere network of regions (i.e., temporo-parietal, occipito-temporal, and inferior frontal) that show differences in structure and/or function between individuals with dyslexia and age-matched controls. This network is reflected in early brain models of reading and reading disability in which temporo-parietal regions are involved in grapheme-phoneme conversion, occipito-temporal regions are involved in word-form identification, and inferior frontal regions are involved in articulatory output (Pugh et al., 2000, 2001). These core attributes still provide the basis for more recent (and more complex) models of reading where the relative contribution of brain regions to reading and language is described with greater granularity (Dehaene, 2009; Price, 2012).

While this general left hemisphere dominant model is prominent (and many results are interpreted in the context of this left hemisphere deficiency model), there is not necessarily consensus among studies as to the contributions of individual brain regions to dyslexia (for example, a large study across multiple countries and languages where the only group difference was the thalamus; Jednoróg et al., 2015). There are likely several complicating factors that contribute to variable results observed across individual studies. For example, the age of subjects is likely to play a substantial role in the heterogeneity of results (Richlan et al., 2011; Black et al., 2017). Studies often use an age range in both their typical reading controls and individuals with dyslexia that include beginning readers (i.e., age 6) through adolescents (i.e., age 14+). Although larger samples helps increase statistical power, such wide age ranges presume the profile of dyslexia is invariant to age and experience (Black et al., 2017). This relates to the idea that older children with dyslexia are likely to have received increased instruction and/or intervention, or at the very least have acquired additional experience with reading. Also, older individuals with dyslexia have also likely had time to compensate for their reading difficulty (Shaywitz et al., 2003). One approach to address this is to focus on studying pre-readers at risk for developing dyslexia (for example, see Raschle et al., 2011, 2012; and recent review: Vandermosten et al., 2016). Following at-risk children into their reading years (and potential diagnosis) allows for the exploration of brain precursors to the disorder (Clark et al., 2014; Kraft et al., 2016). Another experimental approach is comparing both reading-matched controls and age-matched controls to examine the effect of reading experience on brain differences in dyslexia (Hoeft et al., 2007; Krafnick et al., 2014). More such studies, as well as those in larger cohorts with a tighter age range offer promise to help clarify the variability seen thus far.

While there are other issues relating to the variability across studies that will not be discussed here in the interest of scope [e.g., differences in language/orthography (Paulesu et al., 2001; Martin et al., 2015), the role of the cerebellum (Nicolson et al., 2001); non-phonological deficit subtypes/theories (Goswami, 2003; Ramus, 2003, 2004)], a clear major contributor to differences across studies is sex. The contributions of this factor have begun to be explored by the neuroimaging community, which we review next.

### Sex Differences in Dyslexia Brain Research

Dyslexia is ripe for a male-biased literature in part due to the higher odds ratio (Flannery et al., 2000; Katusic et al., 2001; Rutter et al., 2004; Liederman et al., 2005; Quinn and Wagner, 2015). Importantly, this higher odds ratio exists even when controlling for ascertainment bias (Liederman et al., 2005; Quinn and Wagner, 2015). While Lambe (1999) called for studying sex differences in neuroimaging studies of dyslexia nearly 20 years ago, there has been relatively little traction until recently. Studies are much more likely to have balanced inclusion of males and females in their samples today than in the early dyslexia neuroimaging literature, but still suffer from being interpreted in light of models developed from a male biased literature. More studies designed to directly investigate sex differences in the functional and structural neuroanatomy of dyslexia are necessary in order to better understand the impact of sex on the manifestation of the disorder.

While the study of sex differences in the typical population gives us important information that can inform developmental disorders like dyslexia, only studying these differences in dyslexia specifically will provide a full picture. Here, we will examine the seven studies that examined sex differences in the brain structure of dyslexia using MRI (Sandu et al., 2008; Altarelli et al., 2013, 2014; Clark et al., 2014; Evans et al., 2014; Su et al., 2018; see Table 1 for details on individual studies). To our knowledge, these studies represent the whole of the existing sex differences literature for studies in subjects with dyslexia.

**Table 1:** Sex differences in MRI studies of dyslexia.

Article	Subject demographics		Description of dyslexia sex differences
		
	Sex	Age (years)	
Sandu et al., 2008	*Control*: 8M, 10F	*Control*: 13.5	Control females had greater RH GMV, greater WB/LH/RH WMV, and lower GMV/WMV ratio than females with dyslexia, but no significant differences in male comparisons
	*Dyslexia*: 8M, 5F	*Dyslexia*: 13.2	
Altarelli et al., 2013-Study 1	*Control*: 11M, 8F	*Control*: 11.58	Thicker functionally relevant occipito-temporal cortex in female controls compared to females with dyslexia, but no difference in male comparisons
	*Dyslexia*: 10M, 8F	*Dyslexia*: 11.75	
Altarelli et al., 2013-Study 2	*Control (age-matched)*: 7M, 6F	*Control (age)*: 9.75	Thicker functionally relevant occipito-temporal cortex in female controls compared to females with dyslexia, but no difference in male comparisons
	*Control (reading-matched)*: 7M, 6F	*Control (read)*: 6.67	
	*Dyslexia*: 7M, 6F	*Dyslexia*: 9.83	
Clark et al., 2014^∗^	*Control*: 8M, 5F	*Control*: 11.7	Thicker cortex in several regions throughout LH in male controls compared to males with dyslexia, but no differences in the female comparisons
	*Dyslexia*: 5M, 6F	*Dyslexia*: 11.9	
Evans et al., 2014^∗∗^	*Adult Control*: 14M, 13F	*Adult Female Control*: 27.9	Greater GMV in male controls compared to males with dyslexia mostly in regions within the traditional reading network, while female controls show greater GMV compared to females with dyslexia in sensorimotor regions
	*Child Control*: 15M, 17F	*Adult Female Dyslexia*: 34.0	
	*Adult Dyslexia*: 14M, 13F	*Child Female Control*: 9.1	
	*Child Dyslexia*: 15M, 17F	*Child Female Dyslexia*: 10.1	
		*Adult Male Control*: 41.1	
		*Adult Male Dyslexia*: 42.9	
		*Child Male Control*: 8.3	
		*Child Male Dyslexia*: 9.6	
Altarelli et al., 2014	*Control*: 20M, 15F	*Control*: 11.0	Greater rightward asymmetry of *planum temporale* surface area in males with dyslexia, but not in females with dyslexia
	*Dyslexia*: 25M, 21F	*Dyslexia*: 11.0	
Su et al., 2018	*Control*: 11M, 11F	*Control*: 11.1	Greater axial diffusivity of left inferior longitudinal fasciculus in control females compared to females with dyslexia, but no difference between male groups
	*Dyslexia*: 11M, 7F	*Dyslexia*: 11.1	


*^∗^Subject demographics given only for the portion of study relevant to sex differences. ^∗∗^Evans et al. (2014) separately examined males and females (and adults and children) resulting in eight different groups. M, male; F, female; WB, whole brain; LH, left hemisphere; RH, right hemisphere; GMV, gray matter volume; WMV, white matter volume.*

Examining primarily whole brain and whole hemisphere volumes, Sandu et al. (2008) found differences between controls and individuals with dyslexia, with larger effects in females. No significant differences were found in gray matter volume between males and females with dyslexia, but control females had greater volume than those with dyslexia in the right hemisphere, which was not the case in males (Sandu et al., 2008). Males with dyslexia showed greater whole brain, left hemisphere, and right hemisphere white matter volume compared to females with dyslexia (a similar but non-significant trend was found in the control data). Control females showed larger whole brain, left hemisphere, and right hemisphere white matter volume compared to females with dyslexia. Again, males did not show this effect (Sandu et al., 2008). The gray matter/white matter ratio showed significant sex differences in individuals with dyslexia for whole brain, left hemisphere, and right hemisphere measures with females having higher ratios than males (also true for the control group). Females with dyslexia showed higher ratios than control females in the left hemisphere, and this difference was not observed in males (Sandu et al., 2008). Overall, these results indicate volumetric effects at the gross level in females with dyslexia that are not present in their male counterparts.

In two independent studies, Altarelli et al. (2013) used fMRI to identify individual peaks within regions of interest in occipito-temporal cortex that responded to words more than other visual object categories (faces and houses); a standard strategy for identifying the VWFA. These peak locations were used to investigate cortical thickness differences between the age-matched control and dyslexia groups. No sex differences (or interactions) were observed in the location or extent of activation for VWFA brain activity. Both studies showed thicker cortex surrounding these peak functional responses in the age-matched control group compared to the group with dyslexia. Additionally, both studies found group x sex interactions where this effect was only observed in the female subjects (Altarelli et al., 2013). That is, female age-matched controls had thicker cortex around the peak functional responses compared to females with dyslexia, but there was no significant effect in age-matched male controls versus males with dyslexia for either study. The second study also included a reading-matched group of controls to examine how the individuals with dyslexia compare to younger subjects who read at the same level. Using the same size regions as in the age-matched comparisons, there were no significant differences between the reading-matched controls and dyslexia groups. However, when a more focal region of interest was applied (4 mm spheres, as opposed to the 10 mm spheres in the analyses described above), there were again differences between the groups that were specific to females without dyslexia showing thicker cortex than females with dyslexia, but not in the corresponding comparison in males (Altarelli et al., 2013). This technique (use of a reading-matched control group) is used to look for differences that are fundamental to dyslexia as opposed to perhaps being due (at least in part) to reading experience, which is possible when comparing to age-matched controls (Goswami and Bryant, 1989). These results suggest there may be a causal role of cortical thickness in this region for females with dyslexia.

Using a longitudinal design, Clark et al. (2014) examined cortical thickness data acquired in children beginning prior to formal reading instruction (age 6) through potential formal diagnosis of dyslexia (age 11). Sex differences at the pre-reading stage are not described (though it is unclear if they were examined), but sex differences were found at the third stage of data collection (age 11). In the whole group, control children showed thicker cortex compared to those with dyslexia in left hemisphere anterior middle and superior temporal gyri, and left orbitofrontal cortex. When just males with and without dyslexia were compared, these same regions plus the traditional reading network (left inferior frontal gyrus, left temporo-parietal cortex, left fusiform gyrus) also showed thicker cortex in male controls compared to males with dyslexia (Clark et al., 2014). No significant differences were found at this stage between females with and without dyslexia.

Evans et al. (2014) specifically examined volumetric differences between individuals with dyslexia and controls in males and females separately. It is unique compared to the previous two studies in that examining the sexes separately was part of the study design. Four different control versus dyslexia group comparisons were made: female adults, male adults, female children, and male children. In adults: male controls showed greater gray matter volume in left middle/inferior temporal gyri and right supramarginal gyrus, whereas female controls showed greater gray matter volume in right precuneus and medial frontal gyrus/paracentral lobule (Evans et al., 2014). In children: male controls showed greater gray matter volume in left supramarginal/angular gyrus, whereas female controls showed greater gray matter volume in left cuneus and right precentral and postcentral gyrus including the central sulcus (Evans et al., 2014). Together, the pattern across children and adults appears to be that males with dyslexia show less volume in traditional reading network areas, while females with dyslexia show less volume in sensorimotor areas.

Addressing a classic question in the dyslexia literature, Altarelli et al. (2014) examined asymmetry of *planum temporale*, posterior rami, and Heschl’s gyri in children with and without dyslexia. Males with dyslexia showed greater right hemisphere surface area compared to the left hemisphere of the *planum temporale* (overall males had a higher incidence of rightward asymmetry of the *planum temporale* as opposed to the more common leftward asymmetry observed in the greater population; Geschwind and Levitsky, 1968). Males with dyslexia also showed a higher incidence of right hemisphere Heschl’s gyrus duplication compared to control males (Altarelli et al., 2014). There were no significant findings between the female groups for surface area or asymmetry index, and no significant differences for cortical thickness analyses (Altarelli et al., 2014).

In a diffusion tensor imaging (DTI) study of Chinese children, Su et al. (2018) investigated alterations in dyslexia within bilateral arcuate, inferior fronto-occipital, and inferior longitudinal fasciculi. Beginning with fractional anisotropy of these tracts, sex was included in all models but there were no effects of sex or interactions with sex (Su et al., 2018). In further examining the left arcuate and inferior longitudinal fasciculi (where diagnosis effects of fractional anisotropy were observed), there was a diagnosis x sex interaction for axial diffusivity in the left inferior longitudinal fasciculus. Specifically, females with dyslexia showed lower axial diffusivity compared to control females, but there was no difference between the male groups (Su et al., 2018). As the authors note, these findings are close to the Altarelli et al. (2013) cortical thickness differences that were also observed in females but not males (Su et al., 2018). This could indicate both white and gray matter alterations in this region for females with dyslexia, or perhaps an underlying factor that impacts both of these types of measurements. The specificity of the result for axial diffusivity (and not fractional anisotropy) also complicates interpretation (Su et al., 2018). Axial diffusivity has been related to axonal degeneration as opposed to myelination (see Alexander et al., 2007), though how this impacts the results observed here is unclear.

These studies represent the importance of studying sex differences in the dyslexia neuroimaging literature. While they are too few to build a clear picture on their own or conduct a formal meta-analysis, they do provide a starting point for thinking about neuroanatomical sex differences in dyslexia. The results of Altarelli et al. (2014), Clark et al. (2014), and Evans et al. (2014) suggest that perhaps the traditional reading network is impacted in dyslexia to a greater extent in males than in females. Sandu et al. (2008), Altarelli et al. (2013), and Su et al. (2018) suggest that in some regions, females with dyslexia may show more drastic structural differences compared to controls than their male counterparts. It is important to note that there is a lot of variability in these studies in terms of design, age of participants, sample size, natural orthography/writing system of participants, type of brain measure, etc. Each of these may interact with sex, and especially age, which can further complicate the consistency across studies. There may be sex differences in dyslexia observed at certain stages of development/reading experience that are not present at other times. It is also important to note that the sample sizes in these studies are relatively small. While we have focused on highlighting similarities that provide a framework for contextualizing these differences across studies, it is important to clarify the specific differences in these studies are not consistent (due at least in part to the other factors noted above). More and larger scale studies designed to look for sex differences in dyslexia will help clarify how, to what degree, and under what conditions the neuroanatomical correlates of reading difficulty differ between the sexes.

## Genetic Factors

### Brief Overview of Genetic Risk Factors in Dyslexia

In addition to the extensive literature on the behavioral and brain characteristics of dyslexia, the genetic component of reading disability has long been of interest. The scope of this paper will not provide a detailed review regarding the genetics of dyslexia, however, findings relevant to a potential mechanism for sex differences in neuroanatomy will be incorporated (for more in depth coverage, see reviews: Pennington, 1990; DeFries and Alarcón, 1996; Schumacher et al., 2007; Scerri and Schulte-Körne, 2010; Poelmans et al., 2011; Carrion-Castillo et al., 2013). As discussed by Scerri and Schulte-Körne (2010), heritability estimates of reading ability and reading deficits have a fairly significant range from about 30 to 70% (DeFries et al., 1987; Stevenson et al., 1987; Castles et al., 1999; Davis et al., 2001; Gayán and Olson, 2001; Harlaar et al., 2005; Petrill et al., 2006; Bates et al., 2007; Friend et al., 2009), though the ages of participants and the specific reading constructs being measured vary (Scerri and Schulte-Körne, 2010). So while environmental and experiential factors play crucial roles in reading development (which can be observed at the brain level as well; Carreiras et al., 2009; Dehaene et al., 2010; Krafnick et al., 2014), genetic components clearly also have a strong influence on reading ability.

Going deeper than broad heritability, several genes have been identified with mutations that confer risk of dyslexia diagnosis and/or contribute to variation in reading and reading related behaviors (Scerri and Schulte-Körne, 2010; Poelmans et al., 2011; Carrion-Castillo et al., 2013). Across the genes identified, the most common risk factors seem to share a role of contributing to neuronal migration during development (Galaburda et al., 2006; Schumacher et al., 2007; Scerri and Schulte-Körne, 2010; Poelmans et al., 2011; Carrion-Castillo et al., 2013). These results align well with the early neuroanatomical *post mortem* studies of individuals with dyslexia that suggested neuronal migration errors occurred in these individuals (Galaburda et al., 1985; Humphreys et al., 1990). In addition to abnormal asymmetry of the *planum temporale*, they reported cortical ectopias on the surface of the brains of those with dyslexia during their lifetime. More recently, anatomical MRI studies have found a relationship between variation in temporo-parietal brain structure and dyslexia risk genes (Darki et al., 2012, 2014).

Overall, this suggests that at least some of the genetic susceptibility to dyslexia may be related to the migration and organization of neurons during early stages of neurodevelopment (Galaburda et al., 2006). However, it is important to note that there are many genes that have been linked with dyslexia other than those discussed here, including genes not related to neuronal migration (like *CNTNAP2*, which is discussed below; and see Guidi et al. (2018) for the need to develop additional theories).

### Genes With Potential Link to Sex Differences

One consistently identified dyslexia risk gene has particular relevance to the understanding of sex differences in dyslexia: dyslexia susceptibility 1 candidate gene 1 (*DYX1C1*). Disrupting the rat homolog (*Dyx1c1*) with RNA interference (RNAi) produces (1) heterotopias in white matter, (2) over-migration of cortical neurons beyond their target layer of cortex (Wang et al., 2006; Rosen et al., 2007; Currier et al., 2011; Szalkowski et al., 2013), resulting in cortical ectopias similar to those observed in humans (Rosen et al., 2007; Threlkeld et al., 2007), and (3) cortical neurons that incorrectly migrate to the hippocampus instead of cortex (Rosen et al., 2007; Threlkeld et al., 2007). Behavioral deficits also manifest in rats with disrupted *Dyx1c1*, in both acoustic processing and visual attention (Szalkowski et al., 2013). There are similar anatomical and behavioral findings for other dyslexia candidate risk genes, for example, *DCDC2* (rat homolog *Dcdc2*; Meng et al., 2005; Burbridge et al., 2008; Gabel et al., 2011; Wang et al., 2011; Adler et al., 2013) and *KIAA0319* (rat homolog *Kiaa0319*; Paracchini et al., 2006; Threlkeld et al., 2007; Peschansky et al., 2010; Szalkowski et al., 2012; Adler et al., 2013; Centanni et al., 2014a,b), however, *DYX1C1/Dyx1c1* is of particular interest for potential sex differences in dyslexia because of its relationship with estrogen.

One study demonstrated an interaction between *DYX1C1* protein and the estrogen receptors alpha and beta (ERα and ERβ) in human and non-human mammal cell lines, as well as *in vivo* for rat homolog *Dyx1c1* in the hippocampus (Massinen et al., 2009). In a follow-up set of *in vitro* experiments, the estrogen receptor ERβ interacted with the *DYX1C1* gene, specifically in a location that includes one of the common single nucleotide polymorphisms (SNPs) associated with risk for dyslexia (rs3743205; Tammimies et al., 2012). When estrogen was present (17 β-estradiol), it increased the expression of *DYX1C1*, providing a possible connection between hormone signals and this dyslexia candidate risk gene (Tammimies et al., 2012). This may suggest a neuroprotective mechanism in females, though these are *in vitro* results and should be interpreted with appropriate caution. Also of interest is a German study (366 family trios; 66 female indices) that found a three SNP haplotype (including rs3743205) conferred risk of dyslexia in females, but not males, in their sample (Dahdouh et al., 2009), suggesting the possibility of differential risk factors for males and females. Further investigation is necessary to elucidate the contribution of *DYX1C1* to dyslexia risk; specifically, additional *in vivo* studies focused on characterizing the relationship between estrogen and *DYX1C1* function and expression would provide stronger evidence of this putative mechanism for sex differences in dyslexia.

Recently, the contactin-associated protein-like 2 (*CNTNAP2*) gene has been found to have sex-specific links with dyslexia in a Chinese population (Gu et al., 2018). Specifically, two SNPs (rs3779031 and rs987456) contained alleles that increased risk of dyslexia in female, but not male subjects in a sample of 726 students aged 6–15 years old (372 dyslexics-90 female, 354 non-dyslexics-87 female; Gu et al., 2018). Adding this to the *DYX1C1* findings described above suggest females with reading difficulty may have unique risk factors compared to males. It also expands the genetic sex differences in dyslexia beyond cortical migration errors and adds to the expanding knowledge of how dyslexia may develop.

To our knowledge, these are the genes that have shown direct links with sex effects in dyslexia. However, it is possible that other dyslexia risk factors interact with sex as well. Further research into dyslexia risk factors and whether there are sex-specific effects is warranted to more fully complete this picture. Regardless, given the sex difference in incidence and recent evidence of brain sex differences discussed above, genetic studies like these provide a potential mechanism for how these differences might arise. Combined with the recent behavioral and anatomical data, we can start to build a model for how sex differences in dyslexia might manifest in the developing brain.

## Conclusion and Future Directions

**FIGURE 2:**
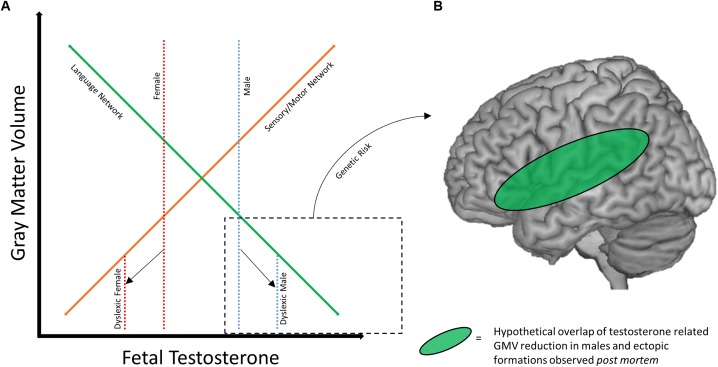
Framework for sex differences in dyslexia brain anatomy. **(A)** Hypothetical relationship between gray matter volume and fetal testosterone. At baseline, males (dotted blue line) may have greater volume of sensory/motor structures (solid orange line) and lesser volume within traditional language network regions (solid green line) compared to females (dotted red line), mediated by fetal testosterone. Adapted with permission from doctoral dissertation (Evans, 2013). **(B)** Hypothetical overlap between volumetric reductions the language network in males with ectopias observed *post mortem*. Reduced GMV and neural migration errors may overlap in the language network of males with dyslexia.

With the existing literature on sex differences in dyslexia in mind, we propose a potential framework for how these differences may develop. Of note, the possibility that deficits in the traditional language network may better characterize males with dyslexia (Altarelli et al., 2014; Clark et al., 2014; Evans et al., 2014), while females instead show aberrations to sensorimotor areas (Evans et al., 2014), is particularly interesting when considered alongside work examining the impact of fetal testosterone levels on adolescent gray matter volume (Lombardo et al., 2012). In a sample of pediatric neurotypical males (8–11 years old), higher levels of testosterone (as measured *in utero*) were correlated with greater gray matter volume in bilateral somatosensory and motor regions, and lower levels of testosterone were correlated with less gray matter volume in bilateral temporo-parietal and frontal regions (including the left hemisphere language network). An overlap of these findings was observed in a cohort of males and females directly compared in an analysis of sexual dimorphisms of gray matter volume (Lombardo et al., 2012). These results suggest that males may confer greater risk for disruption of the reading brain network, which could in turn be exacerbated by fetal testosterone levels. Females, on the other hand, may show similar susceptibility to disruption of sensorimotor brain regions, while the reading network is protected to a greater extent. Figure 2A depicts a hypothetical relationship between gray matter volume, fetal testosterone, and dyslexia. While this relationship is expressed in regards to gray matter volume, the results of Clark et al. (2014) suggest a similar relationship may exist for cortical thickness as well. However, future studies investigating the longitudinal relationship between cortical thickness and hormones are necessary to broaden the model to include cortical thickness as there may be both unique and similar relationships among these variables. The protection of the female brain in language relevant regions may also help explain why females with dyslexia show relatively stronger language skills compared to their male counterparts (e.g., Berninger et al., 2008).

Genetic risk also may mediate or compound the effect of sex/hormones on these brain networks. Returning to the early *post mortem* studies, the pattern of ectopias appears to differ across male and female subjects. Males manifest with a fairly consistent presentation of ectopias in perisylvian structures (Galaburda et al., 1985), while this is not true for the female subjects (Humphreys et al., 1990). Animal models of these ectopias (induced by a freezing probe; Humphreys et al., 1991) also suggest differences between sexes, as males and testosterone treated females show greater anatomical effects of the freezing probe application than do untreated female rats (Rosen et al., 1999). Therefore, it is possible that the anatomical profile of dyslexia genetic risk factors may differ based on sex. Figure 2B diagrams how sex/hormone susceptibility of perisylvian regions in males may overlap with the localization of ectopias caused by genetic risk factors. This helps visualize how a male-dominated literature might identify strong deficits in the anatomy of the reading network, and why males might be more susceptible to reading difficulty in general. Variation in fetal testosterone may affect reading ability as reflected in behavioral differences between the sexes, and in combination with certain genetic risk factors (like *DYX1C1*) could compound potential poor outcomes.

For females, the relative protection of the reading network has important implications for understanding the brain profile of dyslexia. First, it could indicate an entirely separate network of aberrations in females with dyslexia (e.g., Evans et al., 2014). Dyslexia as being the result of sensorimotor impairment (as opposed to a phonological deficit) has been suggested in the literature (Ramus, 2003), though not specifically as an explanatory account specific to females with dyslexia. Second, it could mean that there is more variability in the anatomical profile of dyslexia in females, which might explain the male-only findings of Clark et al. (2014). Third, it is also possible that some females with dyslexia manifest with disruptions within the traditional left hemisphere language network, but perhaps these individuals present with the less likely case of multiple risk factors (e.g., a more masculine brain combined with genetic risk), which would be consistent with the findings of Sandu et al. (2008) and Altarelli et al. (2013). Any one of these scenarios underscore the importance of not assuming that brain-based models of dyslexia formed from male-dominated samples can be directly applied to female or mixed-sex samples.

The potential of sexually dimorphic anatomical profiles of dyslexia due to differential genetic effects is complicated by the fact it is unclear whether there is a difference in heritability between the sexes. Harlaar et al. (2005) found evidence of sex differences in genetic influence on reading disability, which was not replicated in a later study (Hawke et al., 2007). However, even if there is no sex difference in the heritability of reading disability, this does not necessarily mean the anatomical and/or behavioral impact of the genetic risk factors are identical. The estrogen specific *DYX1C1* effects (Massinen et al., 2009; Tammimies et al., 2012), and different risk factors for females provide evidence for this being a real possibility. The genetic profile of dyslexia is complex, and no one gene is the driving factor behind reading difficulty. *DYX1C1* and *CNTNAP2* may only be two examples of potential links between sex differences and the brain in dyslexia.

Focusing on sex differences in dyslexia does not come without concerns and challenges. Sample sizes in dyslexia neuroimaging studies have been relatively small (though no different than neuroimaging studies in general). A recent, large (*n* > 5,000), single-site study in healthy adults found structural and functional sex differences throughout the brain (Ritchie et al., 2018). However, many of the volume, area, and thickness differences were greatly reduced in terms of effect size (or statistically insignificant) when controlling for overall brain size (Ritchie et al., 2018). This highlights the need for larger sample sizes to correctly identify small but true sex differences and eliminate false positives. The existing evidence that we discuss here needs replication to confirm the specific sex differences relevant to dyslexia. Investigators should also report differences before and after controlling for total brain size.

While sex differences may be small, this does not necessarily imply they are inherently not meaningful. It is also possible that there are larger sex differences within the context of disorders or LDs. If this is true, it has meaningful impact on diagnosis and treatment. For example, if there are different pre-reading profiles for at-risk males and females, screening for reading difficulties based on the average will likely miss individuals of both sexes. Similarly, different brain-based manifestations of dyslexia between the sexes may indicate the need for different remediation strategies. As such, neuroimaging studies of reading interventions have also shown variable results (Barquero et al., 2014). Small sex differences in the adult population also do not translate to pediatric populations. Sex differences in brain maturation (Giedd et al., 1999) suggest that the differences between male and female brains (when examined cross-sectionally) may change over time, which can also impact the examination of sex differences in developmental disorders and disabilities.

It is also worth noting the relatively smaller body of evidence thus far for behavioral sex differences in the context of dyslexia. While more examples may come, it is also possible that for males and females with dyslexia, similar behavioral profiles arise via distinct neurological pathways. Whether or not this is the case is worth further examination in both the behavioral and imaging dyslexia literature. Moving forward, studies should not simply control for sex with equal recruitment, but include the direct examination of sex differences as an aim whenever possible. Fully understanding the impact of sex differences in dyslexia requires investigation beginning in pre-readers, continuing after diagnosis, as well as pre- and post-intervention. Thorough investigation across the developmental time course is necessary. Sample size should also be a consideration, as mentioned above. Not finding sex differences in a small to moderate sample does not necessarily mean males and females with dyslexia are the same. While null results may be of concern, the movement toward open science helps alleviate this issue. Many journals now have options for registered reports^2^, where methodology and analysis plans are reviewed and accepted prior to results being known. This has also been advocated for recently by Ramus et al. (2018). Well-designed studies to investigate sex differences in dyslexia are needed regardless of outcome. Understanding when sex does not play a role is just as important as understanding when sex does play a role in dyslexia. The sex differences findings in dyslexia thus far need to be replicated and extended in larger sample sizes. Finally, investigating sex differences in the context of genetic risk factors for dyslexia should also provide key information. If these factors interact as suggested by studies of *DYX1C1* and *CNTNAP2*, it may help clarify how sex differences manifest at the brain and behavioral levels.

However, an important concern is the overall mixed (or lack of) evidence for the neuronal migration hypothesis at different levels of research, which underlies the genetic component of this model. A recent review highlighted this fact, and calls for a reconsideration of the neurobiological and genetic basis of dyslexia (Guidi et al., 2018). The etiology of dyslexia is certainly complex and the model presented here is one potential piece of a complicated puzzle. We encourage continued investigation into genetic and neurobiological underpinnings rooted in alternative explanations of dyslexia that can help explain these putative sex differences. In regards to lack of evidence in neuroimaging literature for the neuronal migration hypothesis (as indicated in Guidi et al., 2018), our model does provide testable hypotheses for *DYX1C1* and other candidate genes related to neuronal migration, which will help clarify whether these genes interact with sex in regards to brain phenotypes or not.

Finally, we have focused here on looking for patterns in neuroimaging studies of sex differences with dyslexia and linking this with what we know about genetics and behavior. It cannot be overstated that the literature here is still young. While we have given a potential framework for how sex differences in the brain may manifest (and how they may relate to heterogeneity across imaging studies), it is quite possible more patterns may emerge as the literature here grows. Our model represents one example of how genetic risk, environmental factors, and brain phenotype may interact.

The pathways that lead to sex differences in dyslexia are likely multifaceted and complex, with several different underlying factors possibly resulting in similar reading deficits. However, this issue can no longer be ignored. We underscore the importance of understanding these differences for the field, especially in regards to the potential impact on both diagnosis and treatment of reading disability. More direct investigation of sex differences, and females with dyslexia, is crucial to the goal of fully understanding the etiology of reading difficulty and the manifestation of brain differences relative to typical readers.

## Author Contributions

AK and TE wrote the manuscript and developed the model.

## Conflict of Interest Statement

The authors declare that the research was conducted in the absence of any commercial or financial relationships that could be construed as a potential conflict of interest.

## Footnotes

1 www.ncbi.nlm.nih.gov/pubmed

2 https://cos.io/rr/
